# An Intelligent Directionality System for Hearing Aids Incorporating Deep Neural Networks: Improving Speech Understanding While Preserving Spatial Awareness

**DOI:** 10.3390/audiolres16050132

**Published:** 2026-09-04

**Authors:** Daniel Marquardt, Jinjun Xiao, Al Ganeshkumar, Jingjing Xu, Larissa Taylor, Martin McKinney, David A. Fabry, Achintya K. Bhowmik

**Affiliations:** 1 Starkey Hearing Technologies, Eden Prairie, MN 55344, USAjingjing_xu@starkey.com (J.X.);; 2 Department of Otolaryngology-Head and Neck Surgery, Stanford University School of Medicine, Stanford, CA 94305, USA

**Keywords:** directionality, directionality system, deep neural networks, hearing aids, speech intelligibility, spatial awareness, artificial intelligence

## Abstract

**Background**: Directionality algorithms are fundamental to modern hearing aids, enhancing speech perception in noisy environments by improving the signal-to-noise ratio. Conventional adaptive approaches rely on heuristic optimization criteria and often improve speech understanding from specific directions, typically the front, at the expense of spatial awareness. A deep neural network (DNN)-based directionality system was developed that dynamically optimizes spatial filtering through a data-driven framework capable of representing complex acoustic scenes. The system intelligently enhances target speech while preserving awareness of environmental sounds. **Methods**: The proposed DNN-based directionality system was evaluated against conventional directionality algorithms by assessing word recognition performance, perceived speech clarity, and listener preference across diverse acoustic scenarios. To complement subjective measures, an artificial intelligence (AI)-based objective intelligibility metric was additionally developed using automated speech-to-text analysis, enabling scalable and consistent benchmarking across devices. **Results**: Results across the behavioral and objective assessments indicate improved speech intelligibility outcomes together with maintained access to environmental sounds. Mean SRT50 improved by 1.4 dB relative to legacy directionality for target speech presented at 90° and by 4.7 dB relative to an omnidirectional pattern when frontal target speech was accompanied by a rear interfering talker. Spatial adaptation further improved environmental sound audibility in three out of four conditions and significantly improved the detection threshold for speech presented at 135° by 2.42 dB. **Conclusions**: These results show that the system can preserve speech originating away from the front while using acoustic context and spatial filtering to attenuate competing speech that interferes with a frontal conversation.

## 1. Introduction

According to prospective hearing aid users, the top drivers of hearing aid performance have remained relatively constant: sound quality, preventing loudness discomfort, effective speech understanding in background noise, and the ability to locate sound, i.e., spatial awareness [1].

Modern hearing aids leverage advanced signal processing techniques to help individuals with hearing loss navigate complex and dynamic listening environments [2,3,4]. Among these techniques, directionality algorithms are crucial for improving speech perception in noisy settings by focusing on sounds originating from specific directions, most often in the frontal hemisphere [5,6,7,8]. Conversations in large, noisy groups remain challenging for patients with hearing loss for a variety of reasons. Typically, an impaired auditory system and reduced ability to discern speech from noise content culminate in experiencing increased cognitive load and needing to work harder to “fill in the gaps” during conversation, ultimately leading to fatigue and frustration [9]. Traditional directionality systems rely on real-time signal statistics from hearing aid microphones, but this approach is vulnerable to fluctuations, even in spatially static environments, due to the time-varying nature of sound and the need for rapid adaptation [10,11]. These fluctuations can lead to inconsistent or suboptimal performance. While directional processing remains an effective strategy for improving speech understanding, its reliance on adaptive acoustic estimates can limit performance in complex listening environments. To address these challenges, researchers have increasingly turned to data-driven approaches. More recently, deep neural networks (DNNs) have been introduced into hearing-aid signal processing and have shown promising results for real-time speech enhancement in noisy environments [12,13,14,15]. Fitzgerald et al. [14] reported improvements in speech recognition and everyday-listening ratings with DNN-based processing, although the benefit varied across speech materials and noise conditions. Folkeard et al. [15] directly compared DNN-based speech enhancement with conventional processing under omnidirectional and directional microphone configurations. The combination of DNN-based speech enhancement and conventional beamforming produced the strongest overall outcomes, but the magnitude of the benefit depended on the noise type and target direction. These approaches have primarily focused on single-channel speech enhancement or on using DNN-derived information to control conventional hearing-aid processing. Related work has examined the effects of directional processing on spatial awareness. Although directional microphones can improve speech recognition in spatially separated noise, stronger directionality may reduce access to non-frontal sounds or alter spatial cues [7,16,17] which might be related to the limitations of conventional directionality algorithms in hearing aids. Although single-channel enhancement can reduce noise based on learned spectrotemporal characteristics, it cannot directly exploit the spatial information available from multiple microphones. Multichannel directionality therefore remains particularly important for improving speech intelligibility independent of the position of the target speaker as well as the presence of interfering speech sources from different directions.

DNN algorithms are also well-suited for enhancing directionality algorithms in hearing aids by enabling a more nuanced understanding of acoustic scenes [18]. Through exposure to diverse spatial scenarios during training, the DNN is trained to associate specific spatiotemporal patterns with optimal directional responses, i.e., parameters that can be utilized to optimize spatial directivity patterns [19]. This enables the system to enhance the signal-to-noise ratio (SNR) for the target speech source by generating optimal beampatterns, while also recognizing conditions where no speech sources are active, allowing it to prioritize spatial awareness instead. This data-driven machine learning approach facilitates context-aware adaptation of directionality patterns, improving both speech perception and environmental awareness simultaneously.

To motivate incorporating a deep neural network into directionality algorithms, the operating principles are first summarized, and inherent limitations of conventional signal processing-based filtering schemes are pointed out. Classical directionality schemes, which have been widely adopted for hearing aid processing, rely on linear combinations of a small set of pre-calculated beampatterns, most commonly corresponding to the number of microphones available, e.g., two in each receiver-in-canal (RIC) or behind-the-ear (BTE) hearing aid styles. The solution space is intentionally constrained by limiting the degrees of freedom in how these base beampatterns can be combined. For example, the first-order differential microphone combines an omnidirectional response with a dipole [20], whereas the Elko–Pong formulation uses a forward-facing and a rear-facing cardioid as its basis patterns [21]. In both cases, the system must determine a frequency-dependent control parameter that combines these basic patterns into an “optimal” beampattern, typically defined in terms of minimizing the output power.

A widely used strategy for estimating this control parameter is the normalized least-mean-squares (NLMS) algorithm, which adapts coefficients to minimize the output signal power. However, because output power minimization does not differentiate between desired (speech) and undesired (noise) components, these systems require explicit a priori assumptions about the location of the target speaker to avoid suppressing speech. In hearing-aid applications, the assumed target direction is typically in front of the user, a reasonable but not universally valid assumption, particularly in dynamic real-world scenarios. Consequently, although classical approaches are computationally efficient and effective in many conditions, they fundamentally rely on predefined assumptions about the target speech location, do not inherently distinguish speech from noise, and therefore cannot reliably preserve speech originating from non-frontal directions. Furthermore, their adaptive performance is constrained by the inherent trade-off within the NLMS algorithm between convergence speed and residual estimation error.

**Figure 1:**
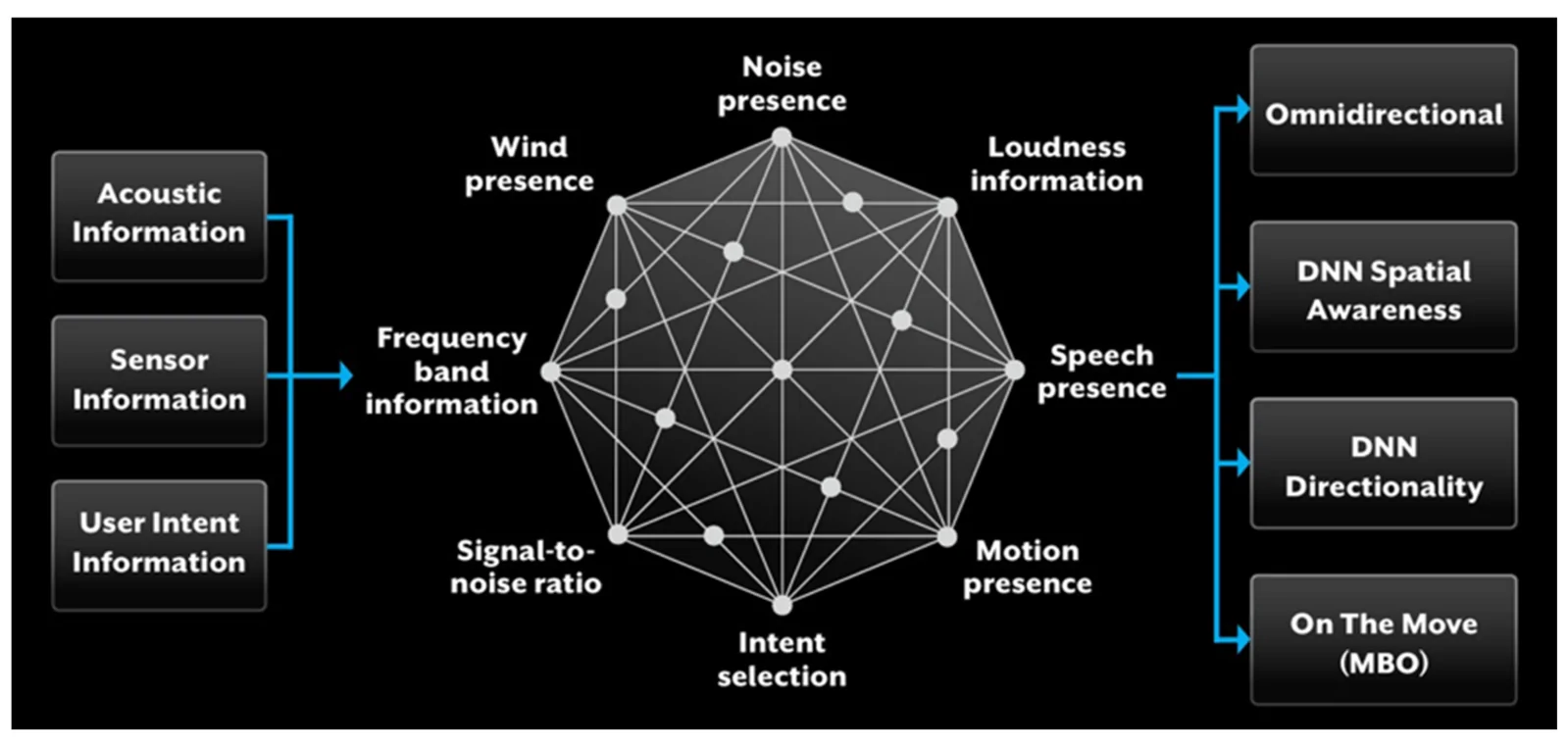
A schematic representation of the intelligent directionality system.

To address these limitations, an intelligent directionality system was developed that addresses this challenge by integrating a wide range of contextual information about the listener and their environment to apply the most appropriate directional settings without compromising the overall listening experience (Figure 1). To evaluate the performance of this DNN-based directionality system vs. the traditional directionality algorithms, a comparative study was carried out to measure the Speech Reception Threshold (SRT), perceived speech clarity, and listening preference for hearing-impaired participants. The assessment involved an adaptive SRT measurement, using the Hearing in Noise Test (HINT), paired-comparison tests for individual preference and perceived speech clarity, audibility of environmental sounds, and evaluating the audibility of spondee words.

Although traditional speech-in-noise tests such as the HINT and the American English Matrix Test (AEMT) are highly valuable for gathering direct feedback from hearing-impaired individuals, the default noise signals used in these tests are stationary, which may not fully represent the complexity and dynamics of real-world acoustic environments. Additionally, behavioral tests are time-consuming and can be cognitively demanding, potentially affecting test–retest reliability. To address these limitations, an efficient, reliable, and scalable approach to acoustic analysis was developed using Speech-to-Text (STT) technology based on artificial intelligence (AI). This method ensures fairness by processing identical input signals across different comparisons, eliminating variability inherent in subjective testing. An automated evaluation is easier to extend to complex acoustic scenarios because the exact same speech and noise signals can be processed by every device. Unlike repeated human testing, different speech lists are not required to reduce learning effects across devices, and dynamic interactions between speech and noise can be retained. For example, a strong transient may have a different effect depending on where it occurs within a sentence, and more natural speech material can be evaluated without substantially increasing the test duration. The approach is also scalable because adding scenarios is not limited by participant time, and it provides consistent scoring that is not influenced by fatigue, attention fluctuations, response variability, or learning effects. These advantages are intended to complement rather than replace behavioral testing.

Previous studies have investigated the relation between objective measures and behaviorally measured speech-intelligibility benefit, e.g., a coordinated series of studies evaluated the same monaural and binaural preprocessing algorithms using instrumental measures, bilateral cochlear-implant users, and normal-hearing and hearing-impaired listeners [8,22,23]. For normal-hearing and hearing-impaired listeners, the binaural speech intelligibility model (BSIM [24]) explained 83% of the variance in individual SRTs in the unprocessed conditions but was less successful in predicting the benefits produced by individual processing algorithms. STOI [25] showed a moderate overall association with behaviorally measured SRT benefit across the tested algorithms and noise conditions, and its predictive performance varied across noise scenarios. More recently, de Oliveira et al. [26] showed that ESTOI, an extended version of STOI [27], predicted improved intelligibility for several speech enhancement algorithms even though behaviorally measured human word recognition declined relative to the unprocessed noisy signals. Modern ASR systems captured this decline more closely and showed strong agreement with relative human recognition trends, although their absolute performance depended on the ASR model and scoring pipeline. These findings provide a rationale for using STT as a scalable objective measure of relative algorithmic benefit while recognizing that the method is not yet sufficiently established to replace behavioral testing.

The purpose of the present project was to evaluate the performance of a DNN-based directional system with respect to speech intelligibility, preference, and spatial awareness. Data from three studies were analyzed to provide a comprehensive assessment. Study 1 examined speech intelligibility and listener preference using comparisons with a traditional directional system. Study 2 evaluated spatial awareness relative to a traditional directional system. Study 3 employed an AI-based speech recognition metric to benchmark the directional performance of the DNN-based system against competing hearing aid directional microphone technologies.

## 2. Materials and Methods

### 2.1. Deep Neural Networks for Directionality

Figure 1 depicts how the directionality system is structured. Information from acoustic and non-acoustic sensors, as well as potential user inputs, is utilized to extract contextual features that are then intelligently combined to determine directional states and optimal spatial filters. The present work focuses exclusively on leveraging the acoustic information within a DNN to obtain the optimal beampattern for directional filtering under the given acoustic scenario. To address previously discussed limitations of directionality algorithms utilized in hearing aids, the proposed approach employs a DNN-based modification of the classical filtering strategy, conceptually similar to that of Elko (2004) [21]. Instead of relying on NLMS adaptation and a priori assumption of the target speaker location, the DNN algorithm learns to estimate the optimal mixing parameter for combining a calibrated front and rear cardioid beampattern directly from the data.

**Figure 2:**
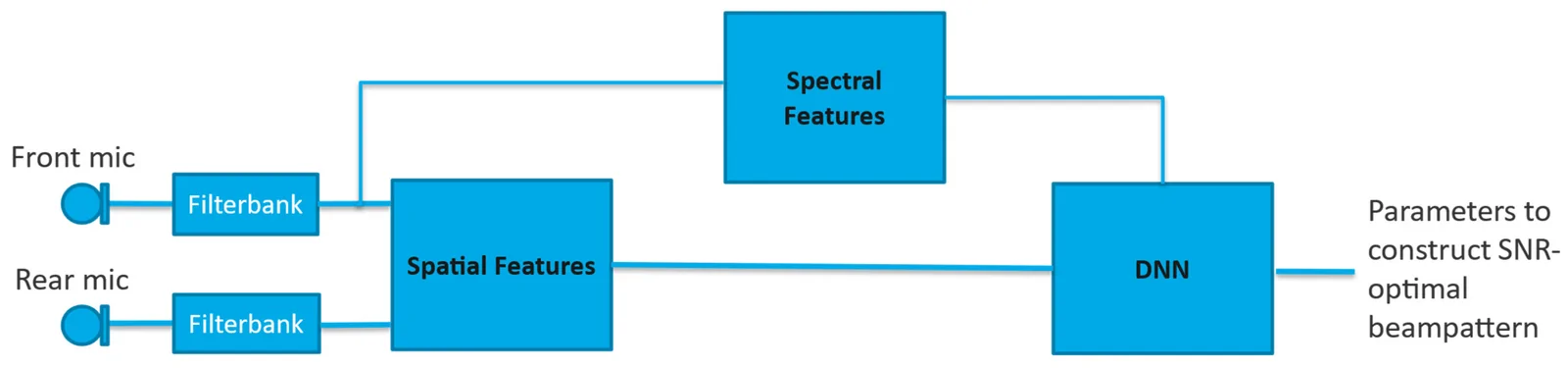
Processing pipeline to generate an SNR-optimal beampattern using DNNs.

The overall algorithm is illustrated in Figure 2. The two time-domain signals from the frontal and rear microphones, spaced approximately 1 cm apart in an end-fire configuration, are first transformed into the frequency domain using a filterbank analysis. From the front-microphone spectrum, spectral features are derived such as log-spectral amplitudes, which are informative for distinguishing speech from non-speech patterns. To capture spatial structure, a normalized cross correlation is computed between the two microphone channels [28], providing information about source direction, early reflections, and the diffuseness of reverberant speech and noise. Together, these spectral and spatial features form the input to the DNN.

A crucial aspect of achieving an optimal and stable performance is the choice of training strategy. In general, DNN-based directionality systems can be trained using either (i) signal-based objectives, where the directionality algorithm is embedded into the loss and optimized directly for metrics such as SNR, or (ii) target-based objectives, where ideal control parameters are precomputed and the network is trained to reproduce them. The latter approach was adopted, where for each training example, an exhaustive search is performed over the mixing parameter to identify the value that maximizes the output SNR, based on the defined target and interfering sounds. These optimal parameters are the training targets, and the DNN is trained to estimate them using a mean-squared-error loss. This strategy provides explicit control over parameter smoothness and prevents abrupt transitions in the beampattern that might otherwise be perceptually intrusive for the hearing-aid user. The directionality system only uses the two available microphone signals of each hearing aid for directional filtering, i.e., does not apply binaural beamforming. Both microphone signals used for directional filtering are located on the same hearing aid. Binaural information is exchanged only to synchronize the processing state between the left and right devices; the contralateral microphone signals are not used for spatial filtering.

The system also incorporates a binaural spatial adaptation mode. This module uses an additional DNN to estimate the probability of speech activity, enabling the algorithm to infer when the user is engaging in a conversation. If no conversation has been detected, the system prioritizes spatial awareness, rather than pursuing the highest possible SNR, by choosing an appropriate fixed beampattern. For stabilization of the processing, information about the current processing state of each device is shared through a binaural link, synchronizing the processing in the left and right hearing aid.

The training data for the DNN-based directionality algorithm were constructed from a diverse mixture of recorded and simulated audio data. In addition to purchased and publicly available speech and noise databases, the training data included extensive inhouse multichannel recordings captured with study devices. Because multichannel training data specific to our hearing-aid microphone configurations are not readily available, device style specific datasets were generated by first measuring anechoic 3D steering vectors for each device type. These measurements accounted for microphone sensitivity variations, device-dependent acoustic characteristics, and inter-subject wearing differences, all of which are essential for ensuring spatial robustness across users and form factors. The steering vectors were then used to render virtual hearing-aid recordings under a wide range of acoustic conditions, including simulated rooms generated with room-acoustics software, as well as complex diffuse(-like) noise fields. Diffuse noise fields were synthesized either by randomly sampling between 6 and 20 of the 3D anechoic hearing-aid impulse responses placed on a sphere and convolving them with noise signals, or by sampling impulse responses to estimate a target coherence pattern and generating noise signals that match this coherence [29]. Real-world recordings collected with our devices were added to further enrich the dataset and ensure coverage of challenging everyday scenarios. Speech in noise mixtures were created over SNRs ranging from −10 dB to +20 dB, providing the DNN with a broad distribution of acoustic conditions representative of real listening environments. The in-house recordings were captured using Starkey Omega AI devices (Starkey Hearing Technologies, MN, USA) with micro-RIC and macro-RIC form factors.

The DNN-based directionality algorithm was integrated into the firmware of the RIC-style hearing aid devices, enabling switching between the legacy and DNN processing modes on a per-memory basis. In addition, the binaural spatial adaptation mode could be activated or deactivated as needed for evaluation. This setup enabled controlled comparative single-blinded studies with hearing-impaired participants.

### 2.2. Study1: Word Recognition and Preference Evaluation

**Figure 3:**
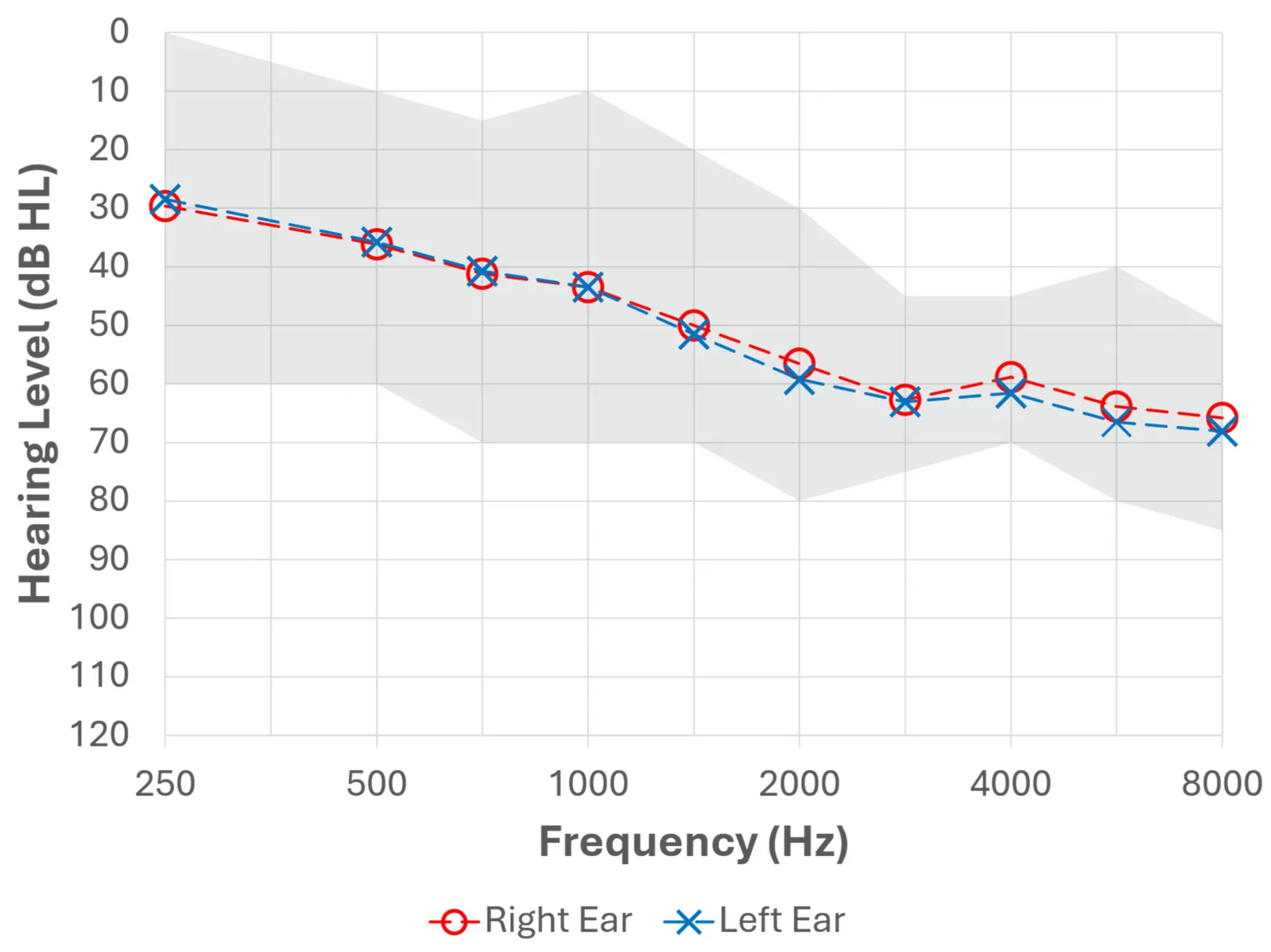
Mean right (red) and left (blue) audiogram and range (shaded gray) of the participants’ hearing thresholds (N = 13) in Study 1.

To measure the 50% SRT, perceived speech clarity, and listening preference of the DNN-powered directionality system, a comparative study was carried out against the legacy classical directionality feature. Thirteen experienced hearing aid users (6 males, 7 females), aged between 71 and 86 years (mean age 77, with a standard deviation of ±5), with symmetrical mild to moderately severe hearing loss (Figure 3), participated in a laboratory study. Participants were fitted with the RIC-style hearing aid devices using occluded power-dome couplings.

#### 2.2.1. Speech Reception Threshold

**Figure 4:**
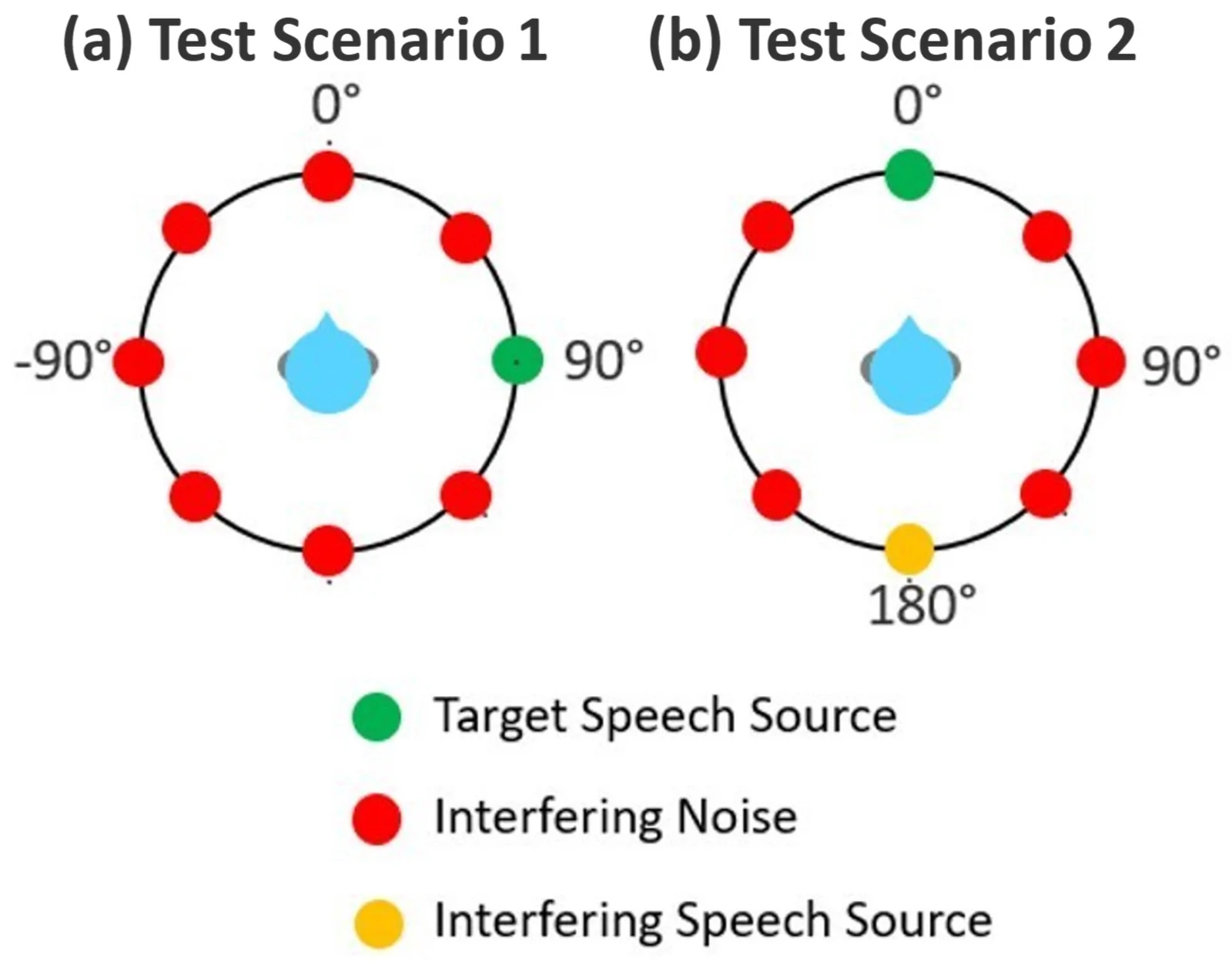
(**a**) Test Scenario 1 laboratory setup with the target speech source at 90°, and (**b**) Test Scenario 2 laboratory setup with the target speech source at 0° and an interfering speech source at 180°.

Test Scenario 1: To demonstrate the newly developed DNN directionality system’s ability to improve word recognition performance for non-frontal target sources, a diffuse babble noise environment was generated using eight loudspeakers arranged in a circular configuration at 45° intervals, each positioned 1 m from the center. The target speech source was placed at 90° (Figure 4a), simulating scenarios such as group conversations, walking in crowded areas, or public transportation.

Test Scenario 2: Traditional approaches to preserving speech from non-frontal directions typically involve switching to an omnidirectional microphone pattern based on a direction-dependent speech-like signal detection mechanism or through user input. However, this approach carries the risk of failing to suppress unwanted interfering signals, such as background conversations or competing speech, which can intrude into the target conversation, particularly in challenging environments like crowded restaurants. To demonstrate that the DNN-based solution offers a more advanced and nuanced approach than simply switching to an omnidirectional pattern, a second scenario was designed that featured the same babble noise used in Test Scenario 1. In this setup, a target speech source was positioned at the front, while an additional interfering speech source was placed at the rear (see Figure 4b). This configuration allowed us to evaluate the system’s ability to effectively manage competing speech signals while maintaining focus on the target speech.

For both scenarios, the noise level was set to 70 dB SPL. For Test Scenario 2, the interfering speech level was set to 75 dB SPL, and for both scenarios, the speech level was adapted according to the HINT procedure to measure the speech reception threshold at which a person can correctly recognize and repeat 50% of the words heard (SRT50). For Test Scenario 1, the DNN directionality system was compared with the legacy directionality, whereas in Test Scenario 2, the DNN directionality system was compared to an omnidirectional polar pattern. SRT50 was selected because the 50% point is a commonly used speech-reception threshold and lies near the steepest region of the psychometric function, providing a sensitive and efficient estimate of changes in speech-recognition performance.

#### 2.2.2. Subjective Preference and Perceived Speech Clarity

**Figure 5:**
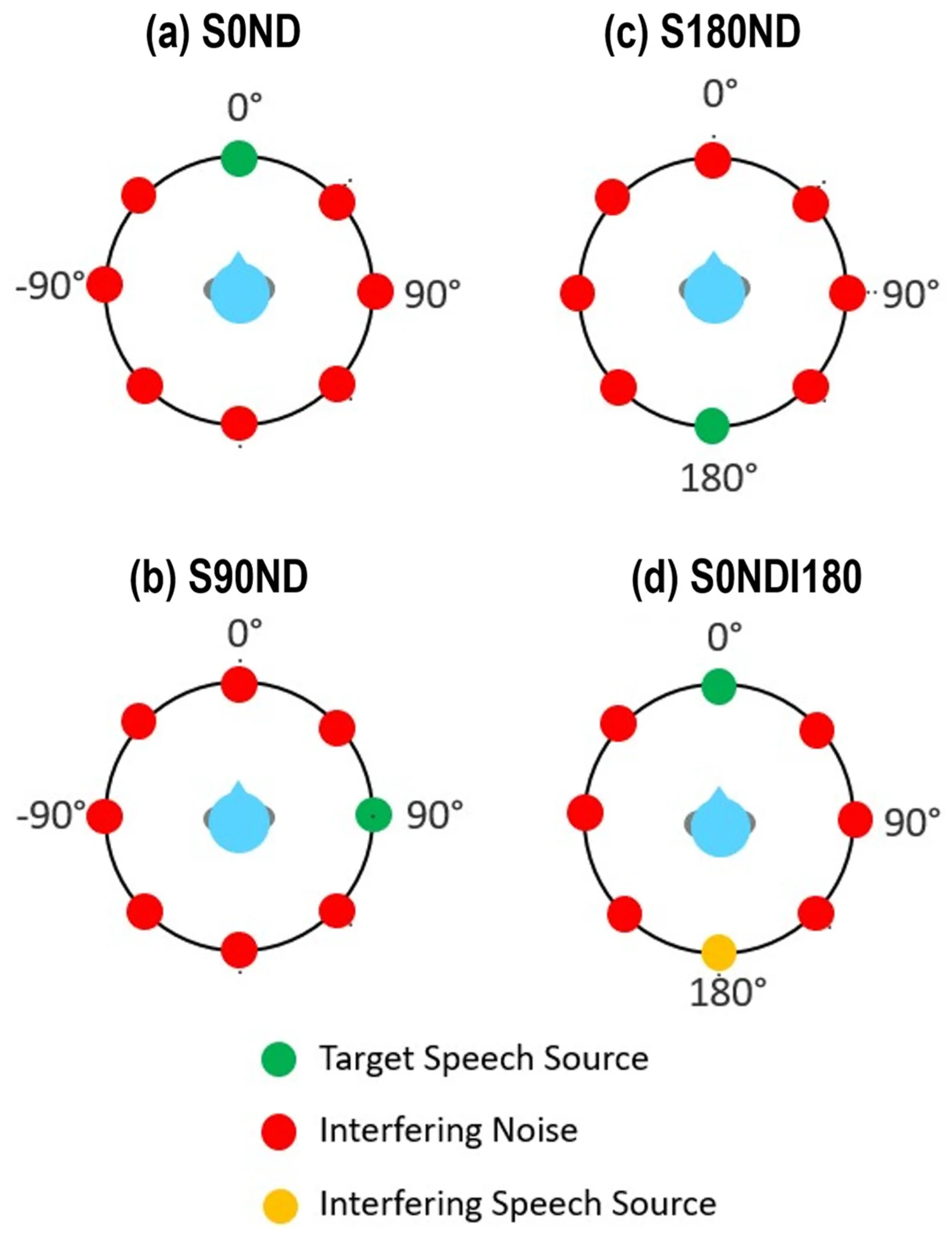
Speech and noise positions for the different acoustic scenarios evaluated: (**a**) female target speech at 0° in diffuse bar noise; (**b**) female target speech at 90° in diffuse city traffic noise; (**c**) female target speech at 180° in diffuse shopping mall noise; and (**d**) female target speech at 0° with an interfering speech source at 180° in diffuse restaurant noise.

While SRT50 measurements are essential to demonstrate the speech perception benefits of the new DNN-powered directionality system, it is equally vital to incorporate complementary subjective assessments regarding personal preference and perceived speech clarity to make sure that the benefit translates into noticeable improvements, beyond standardized test procedures. For this subjective comparison, the following acoustic scenarios were set up (see Figure 5a–d):

S0ND: An 8-speaker diffuse bar noise signal with a female speech signal at 0° (front);

S90ND: An 8-speaker diffuse city traffic noise with a female speech signal at 90° (side);

S180ND: An 8-speaker diffuse shopping mall noise with a female speech at 180° (rear);

S0ND180: An 8-speaker diffuse restaurant noise with female speech at 0° (front) and an interfering speech source at 180° (rear).

Across all acoustic scenarios, the target speech was presented at 71 dB SPL and the diffuse noise at 68 dB SPL (SNR = +3 dB). Where an interfering speech source was included, i.e., S0ND180, the level was set to 75 dB SPL. Audio recordings were obtained via a dummy head with both DNN-based directionality and a legacy directionality system, with processing parameters calibrated to reflect each participant’s individual audiometric profiles. The recordings were then played back to the participant via headphones to allow for a paired comparison between the two directionality systems. For each trial, participants were asked one of the following two questions:

1. Which of the two signals do you prefer to listen to?
2. In which signal does the target speech have the best speech clarity?

The participants could answer, “Signal A”, “Same”, “Signal B”.

In total, there were 8 distinct comparisons (2 different questions, 4 acoustic signals), each repeated 3 times in a randomized order, resulting in a total of 24 comparison trials per participant. To derive a robust representative outcome from the 3 repetitions, a majority vote-filtering method was employed. This approach aggregates repeated responses by selecting the option that received the most votes across the three presentations of each signal. In cases where no option obtained a majority, the aggregated vote was “Same”.

### 2.3. Study 2: Spatial Awareness Evaluation

**Figure 6:**
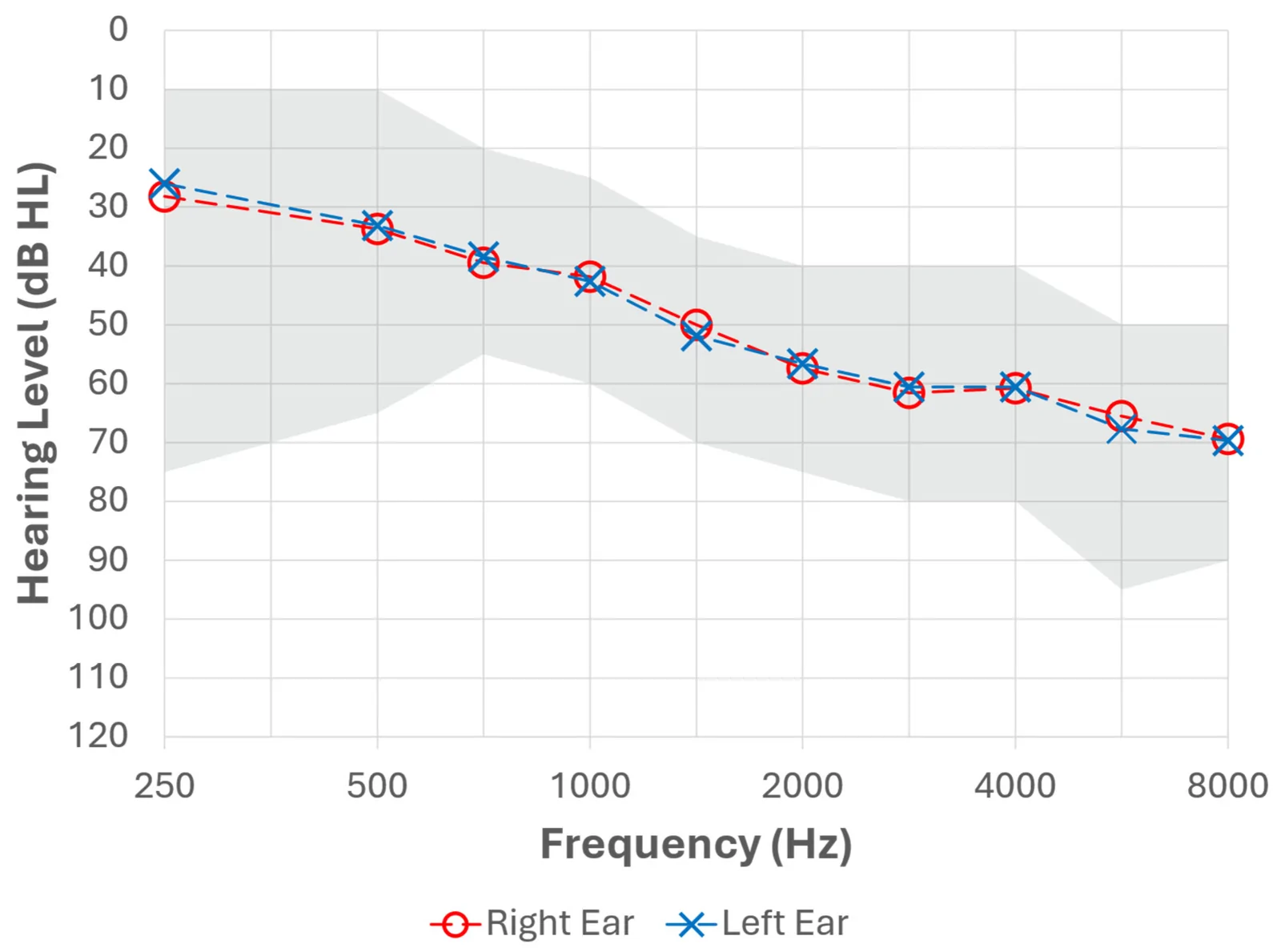
Mean right (red) and left (blue) audiogram and range (shaded gray) of the participants’ hearing thresholds (N = 19) who took part in the study to evaluate spatial awareness in Study 2.

While the use of directionality is a highly effective method to enhance speech understanding, it becomes less desirable in situations where speech is absent. In such cases, excessive directionality can reduce spatial awareness, particularly for sounds coming from the rear hemisphere. This can make it more difficult for listeners to detect important environmental cues, such as a footstep, a doorbell, or a voice from behind. To mitigate these effects, a spatial adaptation mode was incorporated into the DNN directional microphone, with the goal of preserving spatial awareness in situations where speech is absent. Nineteen experienced hearing aid users (11 males, 8 females) with symmetrical mild to moderately severe hearing loss (Figure 6) took part in this study to evaluate spatial awareness using the DNN directionality system. Participants ranged in age from 26 to 82 years (mean age 68.9, standard deviation ± 13.8), and each participant was fitted with the acoustic coupling recommended by the fitting software based on their hearing loss (5 open fittings and 14 occluded fittings).

#### 2.3.1. Audibility of Environmental Sounds

**Figure 7:**
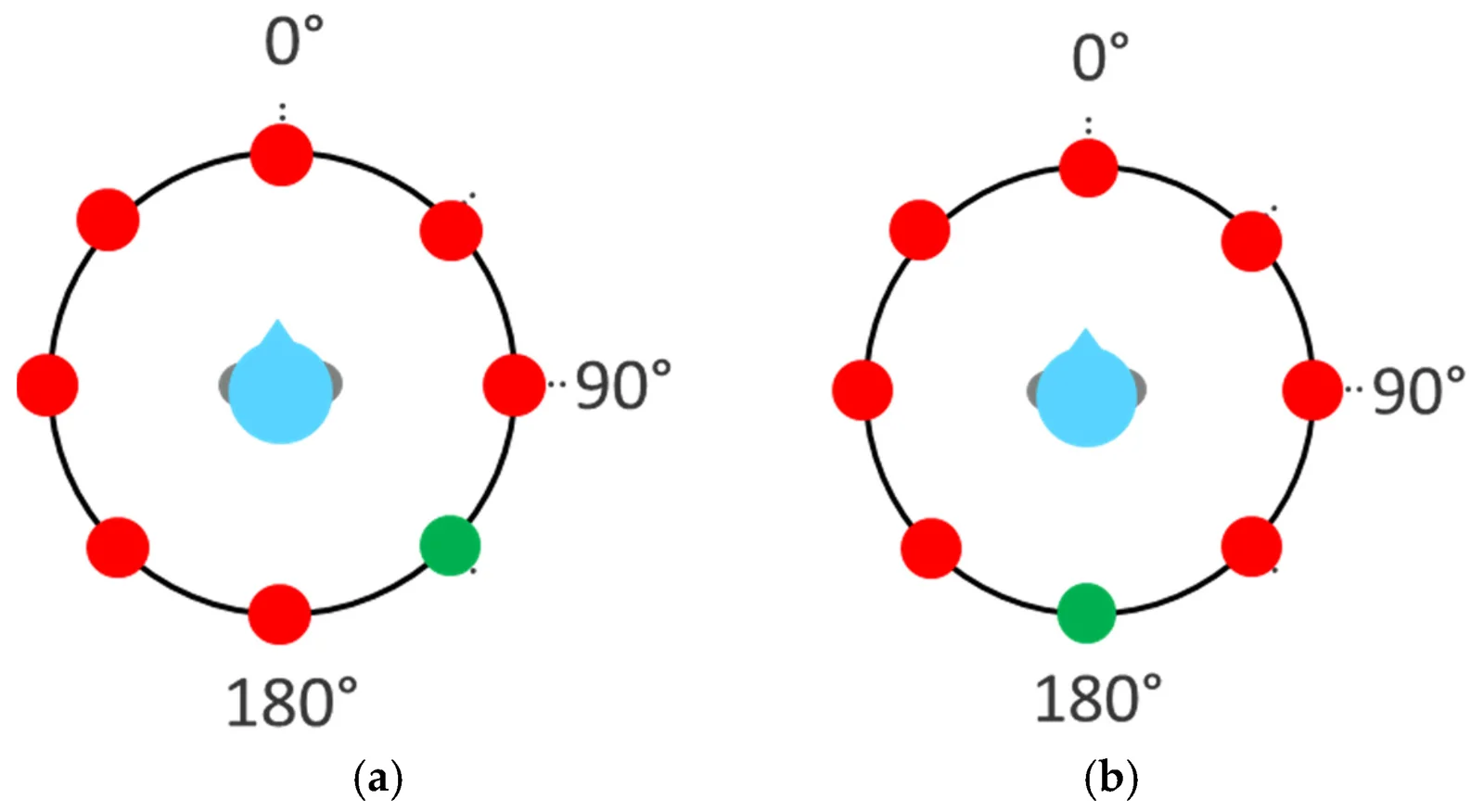
Two test conditions with background noise (red) and target sounds (green) presented at (**a**) 135° and (**b**) 180° azimuth.

In this test, participants compared the audibility of environmental sounds with two microphone settings: (1) a default directional setting and (2) the spatial adaptation mode. The lab setup was as shown in Figure 7: the background babble noise was played at 70 dB SPL, with target sounds presented either at 135° or 180°. Participants were instructed to first find the softest audible level for the target sounds and then compare it between the two settings to indicate which was more audible.

#### 2.3.2. Improved Off-Axis Speech Onset

The DNN directionality system was developed to enhance SNR from the very beginning of speech and throughout ongoing conversations. As previously mentioned, traditional directional processing tends to attenuate sounds from the rear, which can result in the initial onset of speech from behind being suppressed before adaptive systems have time to respond optimally. The evaluation examined whether enabling the spatial adaptation mode improves off-axis speech onset SNR and speech reception thresholds (SRT-50) using two complementary measures: a benchtop SNR assessment and an audibility threshold-in-noise measurement (see detailed descriptions below). These improvements allow listeners to better perceive greetings or speech originating from behind, enabling more natural and timely responses.

##### SNR Measurements

To assess the benefit of the spatial awareness mode for off-axis listening, a benchtop measurement was conducted. In this measurement, the RIC hearing aid device with a DNN-based directionality system was fitted with an N3 audiogram [30] with a power dome. All hearing aid features were at default settings, except for the feedback canceller, which was disabled to accommodate the Hagerman method [31] for SNR calculation. Recordings were made in a sound-treated lab using an 8-speaker array and a Brüel & Kjær Head and Torso Simulator manikin. Food court noise from the ARTE database [32] was played at 70 dB SPL from all speakers, with target speech presented at 67 dB SPL from 135 degrees. The target speech consisted of the short utterances, “heads up” uttered by a female speaker and the utterance “excuse me” uttered by a male speaker. The output SNR was measured with and without binaural spatial adaptation enabled. For the Hagerman phase-inversion procedure, two otherwise identical speech-plus-noise recordings were made, with the phase of the speech signal inverted in the second presentation while the noise was unchanged. Assuming sufficiently consistent hearing-aid processing across the two runs, time-domain subtraction reinforces the speech component and cancels the noise component, whereas time-domain addition reinforces the noise component and cancels the speech component. The separated output components can then be used to calculate objective measures such as output SNR [31].

##### Audibility Threshold in Noise Measurement

In addition, a speech audibility study was conducted to assess the detectability of speech presented from the rear hemisphere (135° and 180° azimuths, see Figure 7) using spondee words. The goal was to determine the lowest sound level at which participants could correctly repeat approximately 50% of the words. Speech materials consisted of 51 spondee words (e.g., “cowboy”, “birthday”, “cupcake”) sourced from the Auditec CD (The Auditec CD Spondees, 2015). Background noise was fixed at 70 dB SPL, while speech stimuli were initially presented at 76 dB SPL (+6 dB SNR). Participants were instructed to repeat each word aloud. A weighted up–down adaptive procedure was used, in which stimulus intensity decreased by 4 dB following a correct response and increased by 2 dB following an incorrect response. The adaptive track was terminated when the listener responded to two consecutive responses at the same level on ascending runs, and this level was taken as the threshold.

### 2.4. Study 3: AI-Based Word Recognition Test

Word or sentence recognition evaluation is an important measure of how well speech is understood and can be assessed objectively or subjectively with human listeners. Although traditional speech in noise tests such as the HINT [33] or the AEMT [34] are highly valuable for gathering direct feedback from hearing-impaired individuals, they come with several limitations. On the one hand, these tests are desirable because they provide insights into how well hearing aid users understand speech under certain conditions, allowing a better understanding of how to address specific needs. On the other hand, these tests typically rely on stationary noise signals like speech-shaped or pink noise, which, while effective at reducing intersubject and intrasubject variability, fail to represent the complexity and dynamics of real-world acoustic environments. Additionally, behavioral tests are time-consuming and can be cognitively demanding, potentially affecting test–retest reliability.

An AI-based word recognition evaluation method utilizing state-of-the-art speech-to-text models offers significant advantages. It ensures fairness by processing identical input signals across different comparisons, eliminating variability inherent in subjective testing, such as differences in hearing loss, individual perceived benefits, and listening environments. Speech-to-text-based evaluation offers several practical advantages as a complement to behavioral speech testing. The same speech and noise signals can be processed by each device or algorithm, enabling direct comparisons without requiring different speech lists to reduce learning effects during repeated measurements. This also allows more complex and dynamic acoustic scenarios to be evaluated. For example, the influence of a transient noise event may depend on its temporal position within a sentence, and such interactions can be preserved by presenting identical recordings to every system. Automated evaluation is also scalable because additional devices, noise types, source configurations, and SNRs can be included without substantially increasing the participant test time. Furthermore, automated scoring is not affected by listener fatigue, fluctuations in attention, response variability, or procedural learning effects.

The proposed system was evaluated against five widely adopted flagship hearing-aid models (RIC form factor) representing major U.S. and European manufacturers available as of the time of the study. The identity of the individual manufacturers was kept anonymous, and the devices are referred to as brands A–E. Each device was fitted bilaterally to the N3 standard audiogram [30] using the respective manufacturers’ proprietary fitting rationale, also known as Best Fit. Power dome coupling was used throughout all devices. Where applicable, “experience level” or acclimatization was set to the maximum (100%) to ensure full algorithmic engagement. All features, e.g., noise reduction, feedback cancellation, remained at the device defaults, i.e., not modifying the settings recommended by the manufacturer. Manufacturers A and B offer a memory-based speech in noise setting, which utilizes DNN processing. For both, the default automatic mode, referred to as A1 (Default) and B1 (Default), and the dedicated DNN mode, referred to as A2 (DNN) and B2 (DNN), were tested, yielding seven comparisons in total.

**Figure 8:**
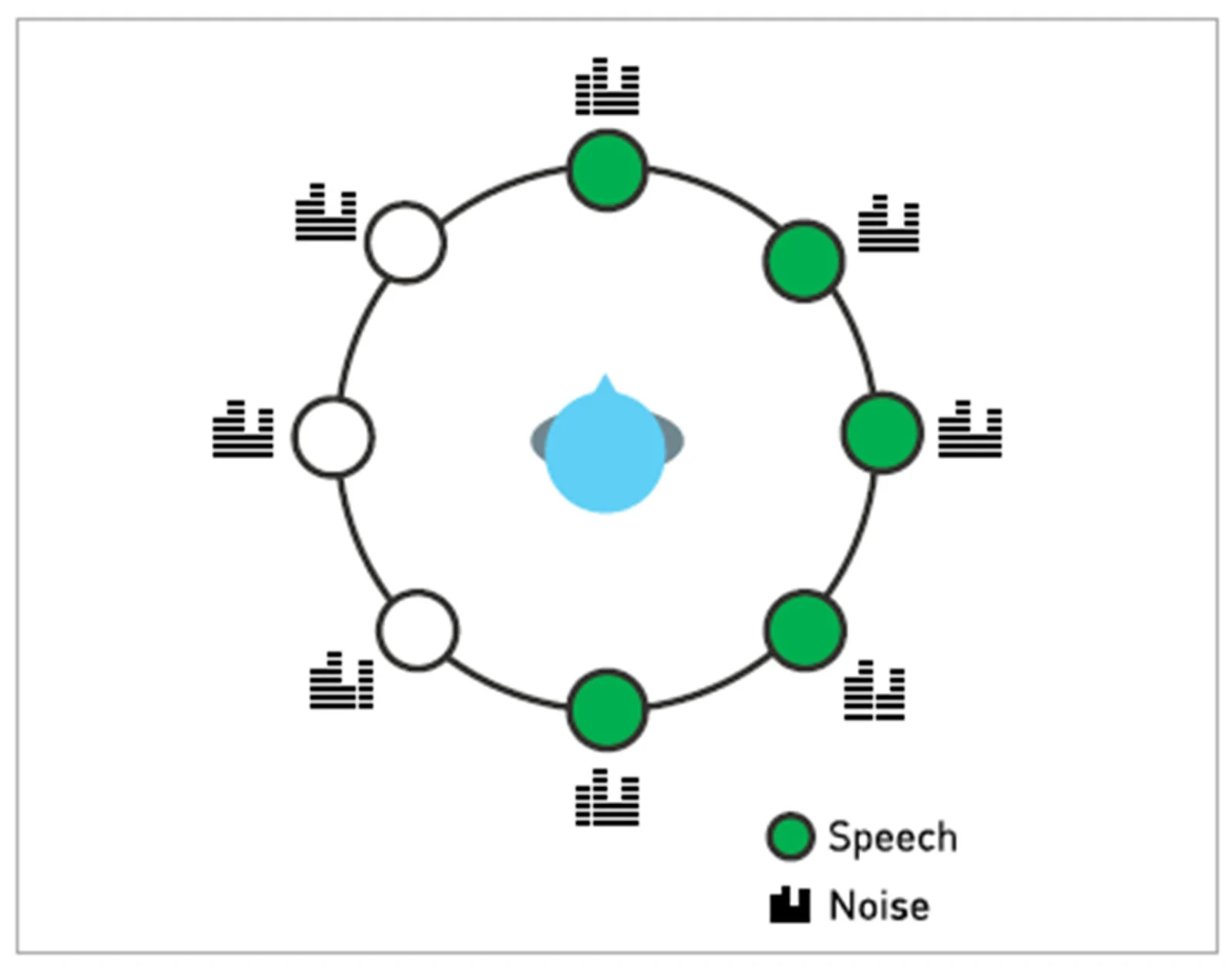
Acoustic scenarios used for evaluation. Location of speech signals used shown as green circles, while diffuse real-world signals (shown as noise signal icon) played through 8 loudspeakers.

A series of acoustic scenarios (see Table 1, Figure 8) was set up within a controlled laboratory environment. These scenarios featured speech and noise signals presented from different locations, with SNRs ranging from −16 dB to 14 dB in 2 dB increments. At each SNR level, 20 sentences from List 1 of the American English Matrix Test (AEMT [34]) were presented [34], with an overall length of 128 s, and incorporated 6 distinct real-world noise files to ensure a comprehensive performance analysis. The device under test was mounted on a Kemar dummy head. The generated acoustic signals were played through an 8-loudspeaker array and the receiver output of the device under test was recorded using the in-ear microphones of the dummy head. It is important to note that for each device, the exact same acoustic signals were recorded. These recorded signals were then used to evaluate the word recognition performance using advanced Speech-to-Text (STT) technology, specifically leveraging the Whisper Automatic Speech Recognition (ASR) system [35]. The “Tiny” model was used to better account for speech perception deficits of hearing-impaired vs. normal hearing. Similarly, as in a word recognition test (like AEMT) every sentence is processed by the ASR to accurately measure the word recognition rates to compare the words detected by the ASR system to the actual words in the sentence. The higher word recognition rate for every sentence between binaural responses was used to account for the better ear effect. Word recognition performance was averaged across the last 10 sentences (leaving the first 10 sentences, i.e., 64 s, for algorithm initialization), producing a single data point for each algorithm in every acoustic scenario, noise type, and SNR. For each scenario and noise condition (30 conditions per device), a sigmoid function was then fitted to the data and the key performance metrics, namely the 50% and 80% Speech Reception Thresholds (SRTs), SRT50 and SRT80, respectively, were extracted, as typically measured in word recognition tests. The SRT50 and SRT80 differences were then calculated between each competitor and the proposed device, i.e., SRT50_competitor–SRT50_Starkey, such that positive values reflect a better performance of the Starkey device. This difference is referred to as “SRT advantage” in the remainder of the paper.

## 3. Results and Discussion

### 3.1. Speech Reception Threshold

First, the results are presented from the SRT50 measurements which have been described in Section 2.2.1.

**Figure 9:**
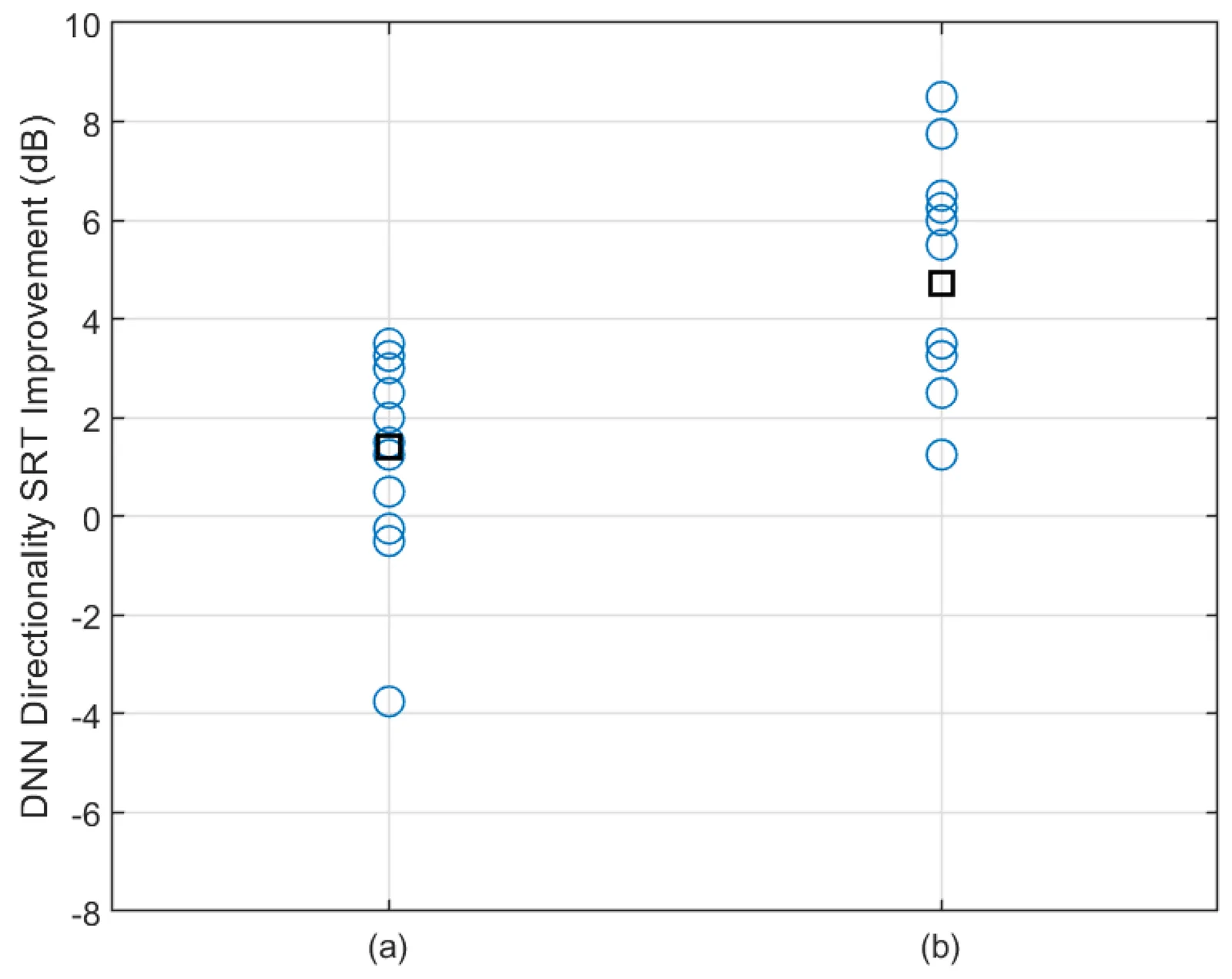
Intelligibility results for the DNN directionality feature compared with (**a**) legacy directionality feature for Test Scenario 1 and with (**b**) omnidirectional pattern for Test Scenario 2. The blue circles represent individual responses while the black squares (□) represent the mean values. More positive values denote better performance by the DNN directionality feature.

For Scenario 1, as shown in Figure 9a, the DNN directionality system achieved a mean SRT50 improvement of 1.4 dB (standard deviation ± 2.0) over a legacy directionality system. A two-sided paired-sample *t*-test was used to evaluate the results, which were statistically significant, *t*(12) = 2.49, *p* = 0.028, with a 95% CI [0.18, 2.63]. The effect size was Cohen’s *d* = 0.69, indicating a medium-to-large within-subject effect.

For Scenario 2, as demonstrated in Figure 9b, the DNN directionality system achieved a mean SRT50 improvement of 4.7 dB (standard deviation ± 2.5) over an omnidirectional pattern. A two-sided paired-sample *t*-test was used to evaluate the results, which were statistically significant, *t*(12) = 6.9, *p* < 0.001, with a 95% CI [3.23, 6.19]. The effect size was Cohen’s *d* = 1.92, indicating a large within-subject effect. Using the established HINT psychometric function slope of 14%/dB for hearing-impaired listeners [36], the SRT50 improvements for Test Scenarios 1 and 2 were translated to estimated intelligibility improvements, which was 19.6% for the first test scenario and 65.8% for the second test scenario. This shows that the DNN directionality system substantially improves speech perception for non-frontal target sources using an intelligent steering design and achieves a better performance than switching to an omnidirectional pattern, as demonstrated in the improvements shown for Test Scenario 2. The two scenarios address complementary aspects of the proposed system. Scenario 1 shows that speech not originating from the front can be preserved more effectively than with the legacy system. Scenario 2 shows that this benefit is not achieved merely by switching to an omnidirectional pattern whenever non-frontal speech is present: when target speech is frontal and competing speech is presented from the rear, the directionality system can utilize learned spatial patterns to suppress the rear interferer. The larger improvement in Scenario 2 is therefore explained by the comparison with an omnidirectional pattern and by the additional directional speech interferer, which provides a greater opportunity for spatial suppression.

Second, the results are presented from the paired comparison study as described in Section 2.2.2.

**Figure 10:**
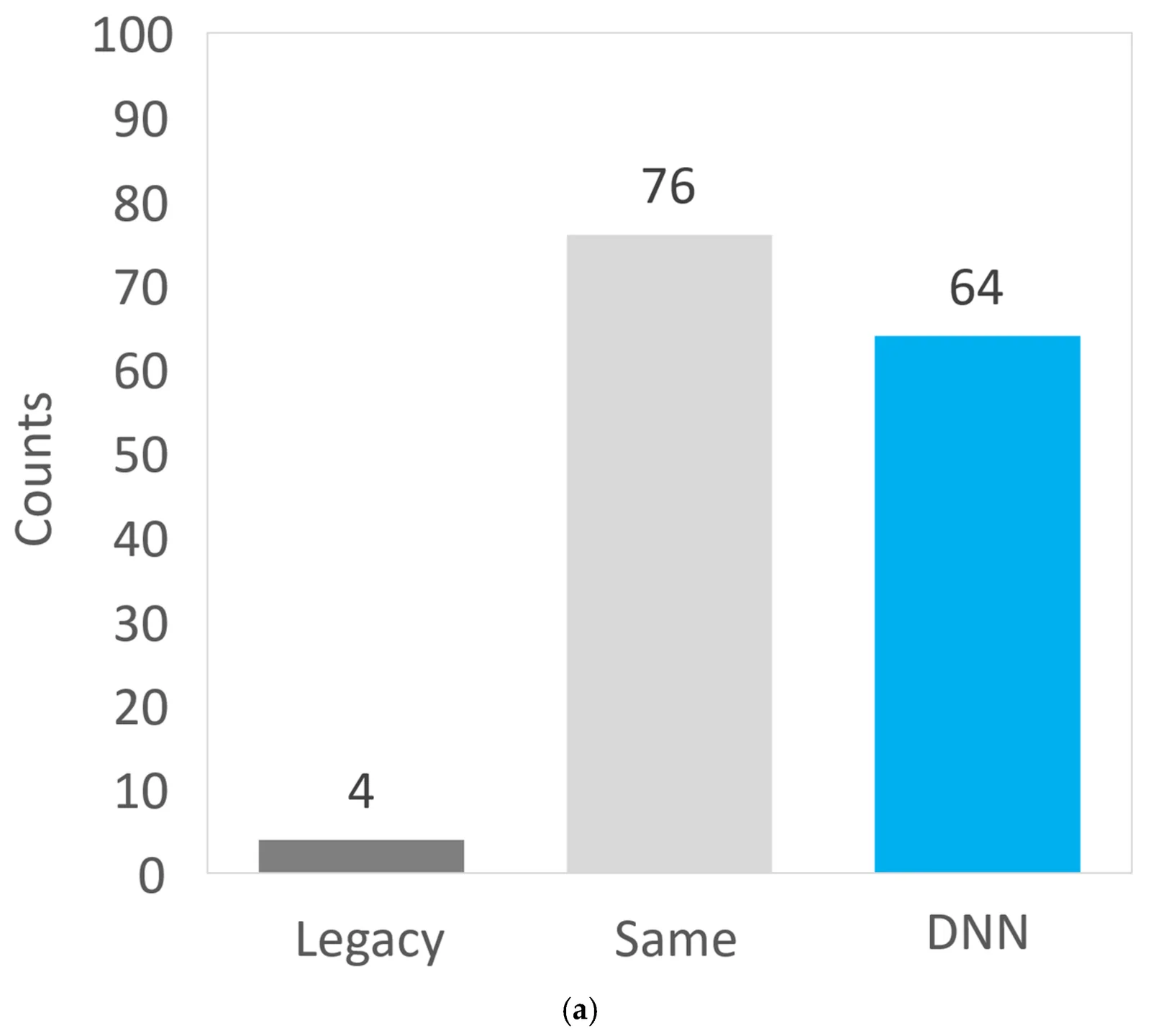

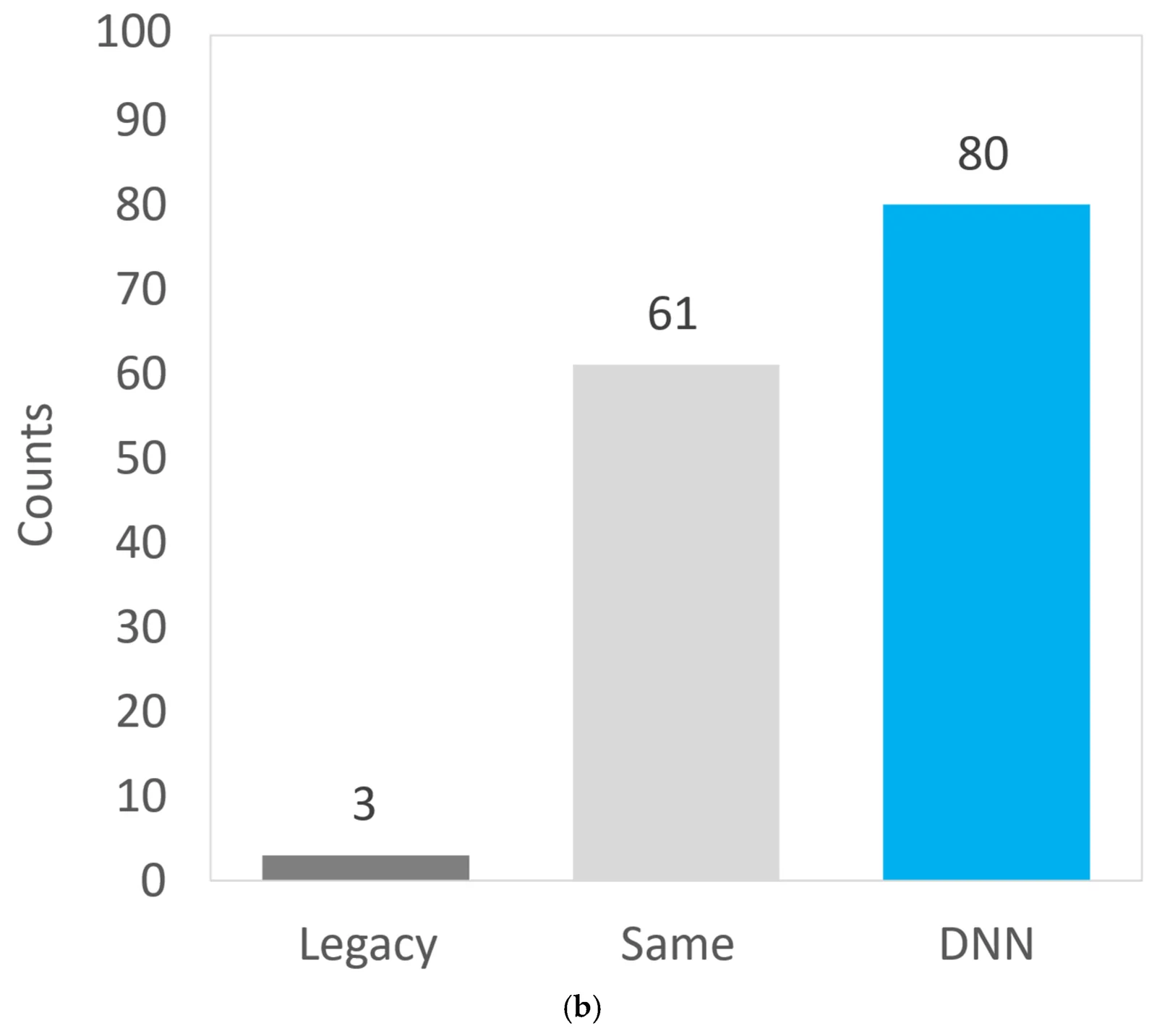
(**a**) Preference for DNN directionality vs. legacy directionality across all acoustic scenarios evaluated. (**b**) Speech clarity for DNN directionality vs. legacy directionality across all acoustic scenarios evaluated.

Figure 10 presents the overall findings from the “preference” and “clarity” evaluations. In the preference assessment shown in Figure 10a, participants expressed a preference in 68 comparisons, of which 94% favored the DNN-based directionality system. Likewise, in the clarity evaluation shown in Figure 10b, participants made 83 preference selections, with 96% favoring the DNN-based system. Although both the “preference” and “clarity” questions point to similar performance differences, a noticeable portion of the original “same” ratings shifted toward the DNN system. This can be explained by the behavior of a suboptimal directionality algorithm: if an algorithm does not achieve SNR-optimal filtering, it often over-suppresses the speech signal. Informal listening confirmed that in some acoustic conditions, the DNN algorithm may provide similar, or occasionally slightly less, noise suppression than the legacy algorithm, but with less speech over-suppression. As a result, speech can sound clearer, even if the additional clarity is not always the dominant factor driving preference, particularly for listeners who may prioritize noise reduction over additional speech clarity.

For statistical analysis, the Bradley–Terry–Davidson model, a trinomial extension of the Bradley–Terry framework that incorporates ties through an additional parameter, was applied [37]. For the preference data, the estimated strength parameters were π_Legacy = 0.25 and π_DNN = 4.00, yielding a strength ratio of π_DNN/π_Legacy = 16, indicating a strong preference for the DNN algorithm when listeners expressed a choice. The estimated tie parameter was ν = 4.75, indicating that ties occurred slightly more frequently than explicit preferences for the DNN system. The log-odds contrast, log(π_Legacy) − log(π_DNN) = −2.77 (95% CI: −3.8 to −1.8), was statistically significant (z = −5.32, *p* < 0.001). The corresponding odds-ratio confidence interval for the Legacy algorithm relative to the DNN system ([0.023, 0.173]) further confirms a large and well-estimated performance difference, showing that in non-tie comparisons, the Legacy algorithm was consistently and substantially less preferred. The proportion of Legacy selections among non-ties was only 0.059 (95% CI: 0.022–0.148), again illustrating preference for the DNN algorithm whenever listeners detected a difference.

For the “clarity” question, the pattern was similar. The estimated strength parameters were π_Legacy = 0.19 and π_DNN = 5.17, resulting in a strength ratio of 27, demonstrating that whenever listeners perceived a clarity difference, they consistently chose the DNN system. The tie parameter ν = 3.94 indicated that ties were less frequent than clarity selections favoring the DNN algorithm. The log-odds contrast, log(π_Legacy) − log(π_DNN) = −3.28 (95% CI: −4.4 to −2.1), was statistically significant (z = −5.32, *p* < 0.001). The corresponding odds-ratio interval for the Legacy system ([0.023, 0.100]) again reflects a large and precisely estimated difference.

In summary, although perceptual differences were often small enough to result in many ties, the Davidson model demonstrates a strong, consistent, and statistically robust advantage for the DNN-based directionality algorithm. Whenever listeners detected a difference, the DNN system was preferred and judged clearer, providing meaningfully improved perceptual performance.

### 3.2. Audibility of Environmental Sounds

**Figure 11:**
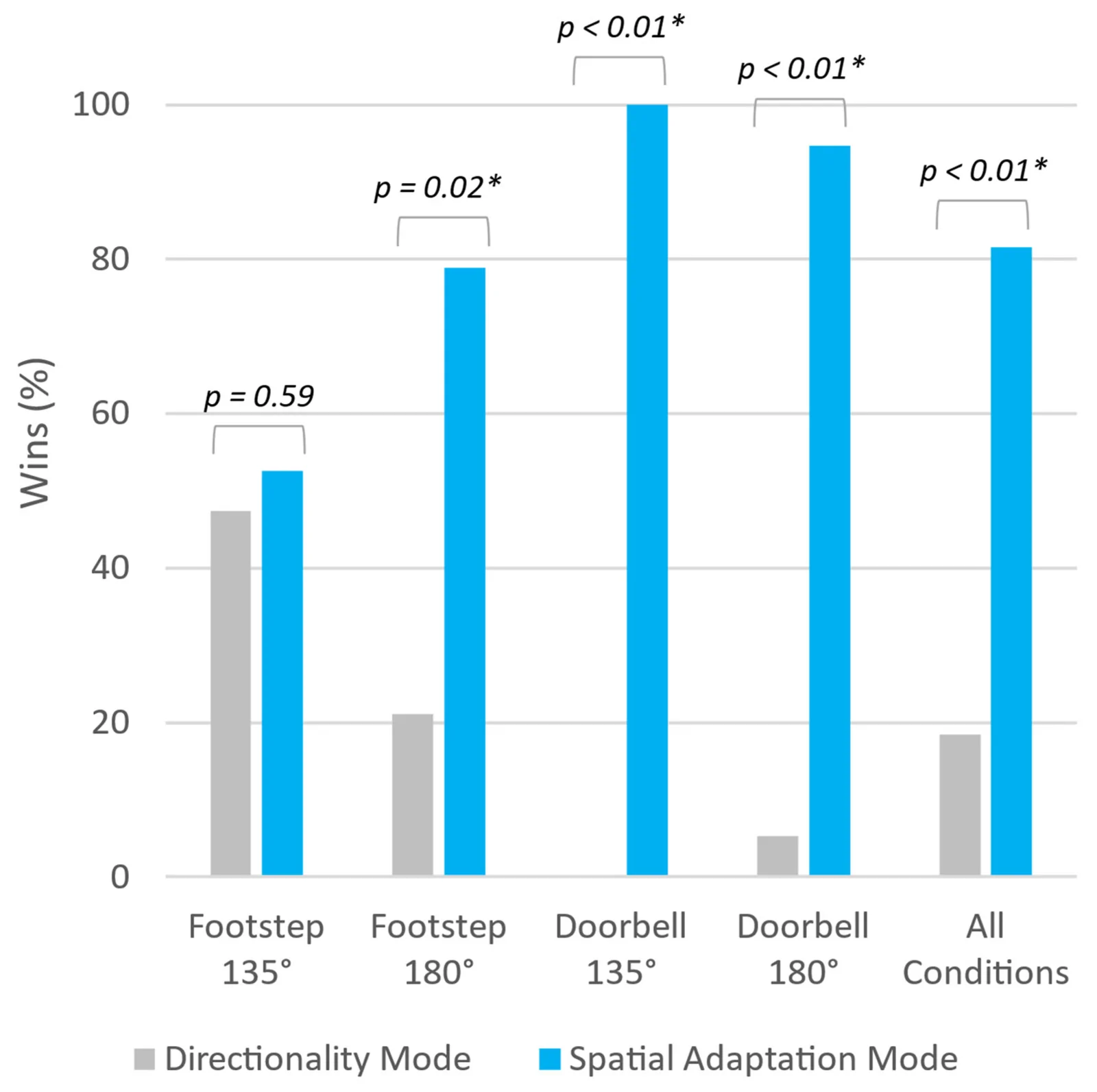
Audibility test results averaged across the two test conditions for the two target sounds. Using the exact binomial test, significant differences were observed (*) in three out of the four conditions, as well as across all conditions tested.

The audibility test results, averaged across the two test conditions for the two target sounds, are shown in Figure 11. Overall, the spatial adaptation mode achieved a higher proportion of wins than the directionality mode in most listening conditions. No significant difference was observed for the Footstep 135° condition (53% vs. 47%; *p* = 0.59). However, the spatial adaptation mode was preferred significantly more often for Footstep 180° (79% vs. 21%; *p* = 0.02), Doorbell 135° (100% vs. 0%; *p* < 0.01), and Doorbell 180° (95% vs. 5%; *p* < 0.01). When data were pooled across all conditions, the spatial adaptation mode accounted for 82% of wins compared with 18% for the directionality mode, a statistically significant difference (*p* < 0.01). These findings indicate that the spatial adaptation mode generally provides better audibility of environmental sounds, particularly for the doorbell stimuli and for sounds originating from the rear hemisphere.

### 3.3. Improved Off-Axis Speech Onset

**Figure 12:**
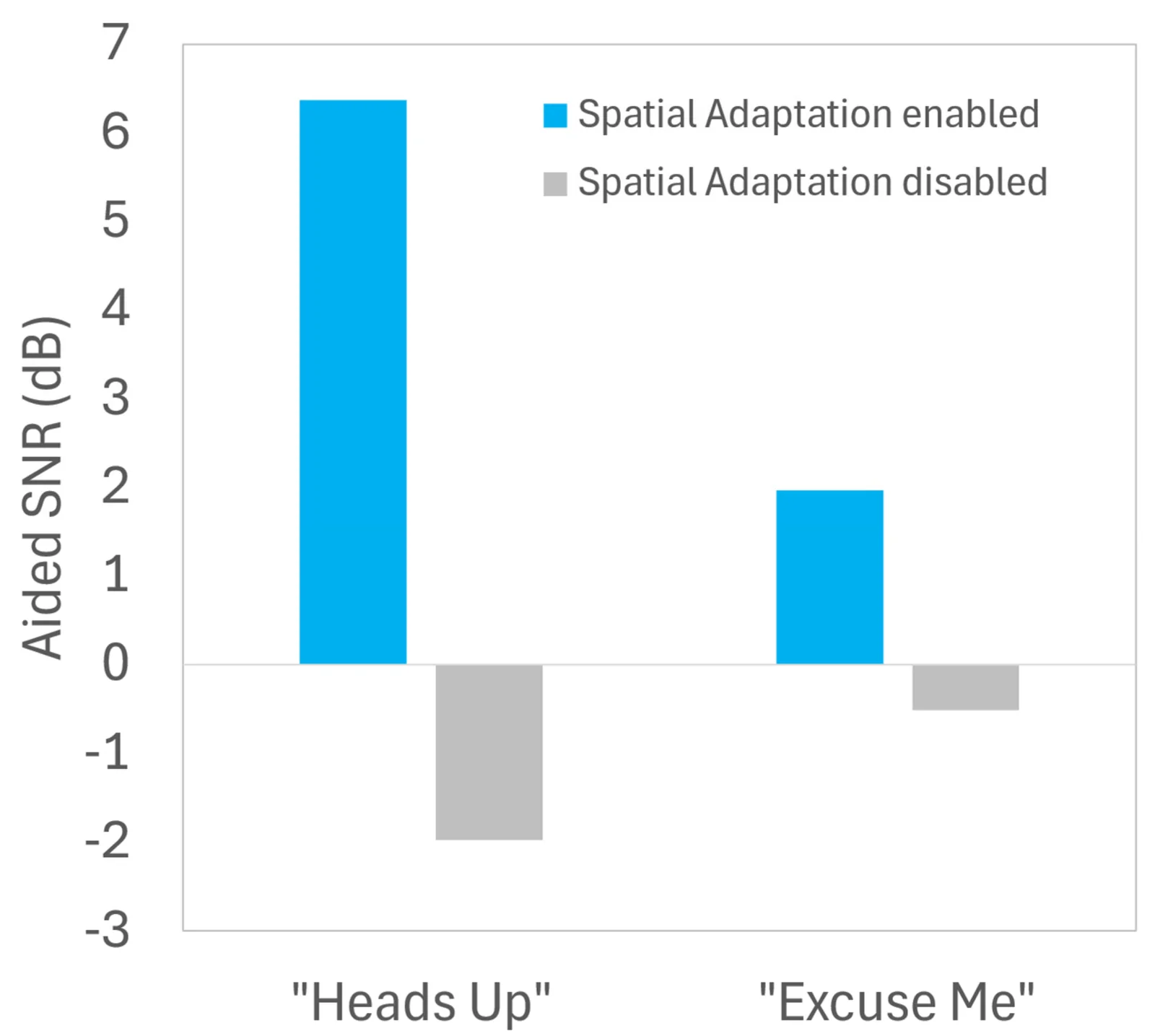
Aided SNR measurement comparing spatial adaptation mode (enabled versus disabled) in two speech conditions.

The results for the SNR measurement described in Section 2.3.2 are shown in Figure 12. Enabling the binaural spatial adaptation improved the output SNR by 8 dB for the female speaker and around 2.5 dB for the male speaker. This indicates that there is a potential to improve the audibility of short utterances coming from the rear hemisphere. The results of this audibility test will be presented in the next subsection.

### 3.4. Speech Audibility

**Figure 13:**
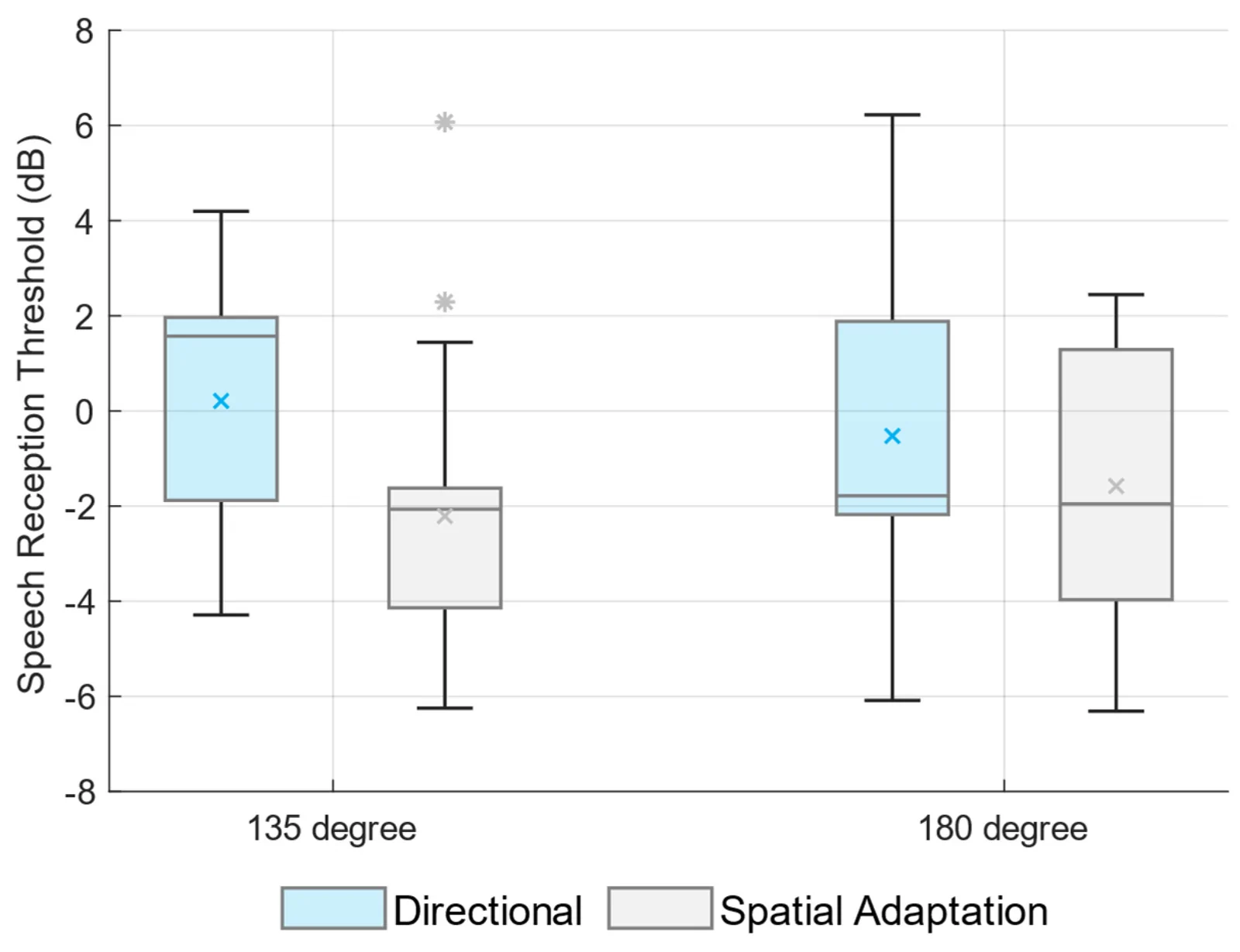
Speech reception threshold measurement comparing directional mode and spatial adaptation mode at 135 and 180 degrees (asterisk *—outliers).

The results for the audibility threshold measurement described in Section 2.3.2 are summarized in Figure 13. Results show that the binaural spatial adaptation mode significantly improved speech reception thresholds compared to traditional directional settings. Specifically, thresholds were lower by approximately 2 dB when speech was presented from the rear (135° and 180°), with mean values of −2.21 dB (SD = 3.11) and −1.58 dB (SD = 2.71), respectively, compared to 0.21 dB (SD = 2.66) and −0.53 dB (SD = 3.26) in directional mode. A two-sided paired-sample *t*-test was used to evaluate the results. For speech originating from 135°, the difference was statistically significant, *t*(18) = −3.41, *p* = 0.003, with a 95% CI [−3.91, −0.93]. The effect size was Cohen’s *d* = −0.78, indicating a medium-to-large within-subject effect. For speech originating from 180°, the difference was not statistically significant, *t*(18) = −1.27, *p* = 0.22, with a 95% CI [−2.79, 0.69]. The corresponding effect size was Cohen’s *d* = −0.29, reflecting a small within-subject effect.

These findings support the efficacy of the DNN directionality system, particularly the use of binaural spatial adaptation mode for enhancing the detectability of brief, off-axis speech signals, although the results for speech presented from 180° was not statistically significant.

### 3.5. AI-Based Word Recognition Evaluation

**Figure 14:**
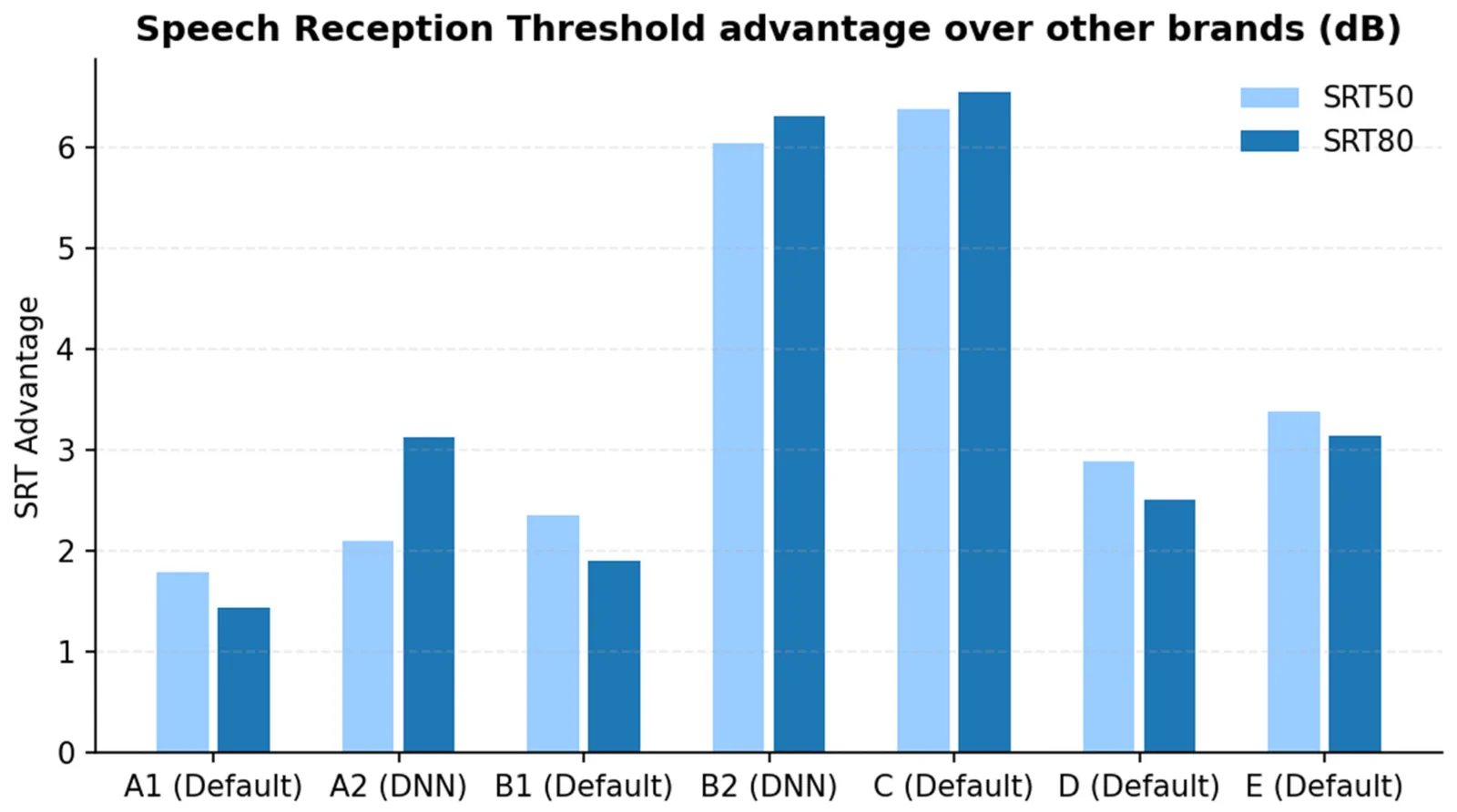
SRT50 and SRT80 advantage of the DNN directionality system over other brands across all of the conditions evaluated.

The performance for the AI-based word recognition test as described in Section 2.4, averaged across all acoustic scenarios as a representation of overall performance, is shown in Figure 14. The bar graph illustrates the SRT advantage in decibels (dB) of the new DNN-based directionality system over various hearing aid models for the two performance measures SRT50 and SRT80. The results reveal notable differences in performance between models and processing strategies. For both, the SRT50 advantage and SRT80 advantage, statistical analyses were conducted using two-sided one-sample *t*-tests (*n* = 30, *df* = 29). To maintain a family-wise error rate of α = 0.05, *p*-values were adjusted within each endpoint using the Holm step-down procedure. Both unadjusted and Holm adjusted *p* values, Cohen’s *d* effect sizes, and 95% confidence intervals for the mean differences are reported. As shown in Table 2, for each measure (SRT50 and SRT80) and for each competitor’s comparisons, the results were statistically significant. Performance varied across manufacturers and processing modes. The reasons for the particularly large SRT advantages, e.g., relative to B2 and C, remain speculative because the underlying algorithms are proprietary. Differences in the directionality strategies used in DNN and non-DNN programs, including whether (fixed) binaural beamforming is activated or deactivated, may have contributed to the observed results. In addition, DNN-based speech enhancement does not necessarily improve speech intelligibility. Enhancement artifacts can reduce both behaviorally measured word recognition and the word-recognition performance of automatic speech-recognition systems [26]. Preserving target speech from arbitrary directions also requires an accurate estimation of the acoustic scene, whether source direction and speech activity are estimated explicitly or inferred indirectly through the processing strategy. Errors in this estimation may attenuate the target speech or preserve an interfering source. These factors may have contributed to the observed differences.

## 4. Conclusions

The present study demonstrates that DNN-based directionality systems can provide substantial improvements in speech perception, spatial awareness, and listener preference compared to conventional directional processing strategies. These findings align with prior evidence suggesting that data-driven spatial filtering can better accommodate dynamic listening contexts than more traditional signal processing-based methods.

The results also demonstrate that speech intelligibility improvements with the DNN-based directionality system can be achieved, while spatial awareness is preserved. Unlike traditional approaches that often sacrifice environmental awareness and audibility for improved speech focus, the proposed system maintains access to non-speech signals through intelligent adaptation. Furthermore, the evaluation of spontaneous word detection confirmed that adaptation occurs rapidly, enabling listeners to respond naturally to unexpected speech from off-axis positions. Although for the preference and clarity test many comparisons resulted in ties, participants strongly favored the DNN-based system for both overall listening preference and perceived speech clarity when they detected a difference between the processing conditions. These findings demonstrate that algorithmic differences were perceptible under the evaluated laboratory conditions. However, further real-world evaluation is required to determine whether these benefits translate into improved everyday listening experiences, user acceptance, and long-term satisfaction.

It should be noted that different acoustic couplings were used across the experiments. The behavioral speech-intelligibility and preference evaluations described in Section 2.2 used occluding power domes, which substantially reduce the direct acoustic path and may increase the measurable effect of directional processing. The manikin-based off-axis speech-onset SNR measurements in Section 2.3.2 also used a power dome with an N3 fitting. In contrast, the environmental-sound audibility test in Section 2.3.1 and the behavioral spondee-word audibility test in Section 2.3.2 were conducted using the acoustic coupling clinically prescribed for each participant. Open or less occluding couplings may preserve additional access to environmental sounds through the direct acoustic path, independently of the hearing-aid processing. Consequently, the speech intelligibility and spatial awareness results demonstrate complementary benefits across the different experiments, but do not establish that both benefits would occur to the same extent under identical coupling conditions or within the same participants.

The introduction of AI-driven word recognition assessment represents an additional contribution of this work. By employing automated speech-to-text analysis, algorithmic performance could be evaluated across a broader range of acoustic conditions without the constraints of cumbersome and time-consuming listening tests, listener fatigue, or restricting evaluations to measurement-like signals. The results show substantial performance differences among manufacturers, highlighting variability in how current systems manage complex acoustic environments. These disparities are likely linked to differences in adaptive processing speed and robustness. In particular, the ability to react rapidly to changes in spatial configuration is particularly important, as, for example, an erroneously steered binaural beamformer can significantly degrade speech intelligibility by attenuating the target signal or amplifying competing sources [38]. The lower Whisper-derived SRTs observed for the proposed system suggest that fast, context-aware adaptation may mitigate these risks within the automated benchmark. Because the metric has not yet been validated against behavioral SRTs in listeners with hearing loss, these relative differences should not be interpreted as equivalent improvements in human speech perception.

Despite these promising results, several limitations warrant consideration. First, the training of DNN models requires extensive multichannel datasets that accurately capture device-specific and user-specific variability. While simulation techniques and ambisonic recordings mitigate this challenge, further work is needed to ensure robustness across diverse real-world conditions. Second, the laboratory-based nature of the current evaluation, although incorporating realistic noise recordings, cannot fully replicate the unpredictability of everyday listening environments. Furthermore, STT-based SRT estimation is an appealing direction for algorithm evaluation, particularly for comparing algorithmic benefits for many conditions and realistic non-stationary noise files. However, to fully understand the predictive strength of AI-based SRT measurements, further studies are needed to establish their validity and to determine how accurately these metrics reflect human listening performance.

## Figures and Tables

**Table 1:** Acoustic scenarios for comparison across brands. The speech signal was played from a single loudspeaker at the respective speech position. The diffuse real-world signals were generated by playing uncorrelated noise snippets through 8 loudspeakers.

	Acoustic Scenarios				
	1	2	3	4	5
Speech Position	0°	45°	90°	135°	180°
Noise Position	Diffuse				
Speech Signal	20 AEMT Sentences				
Noise Signal	Bar Noise, Shopping Mall, Construction, Crowd, Indoor Party, Outdoor				

**Table 2:** Summary of pairwise performance differences across SRT_50_ and SRT_80_. The table reports Cohen’s *d_z_*, 95% confidence intervals of the mean differences, and both unadjusted and Holm-adjusted significance levels.

Comparison	Metric	Cohen’s *d_z_*	95% CI (Mean Diff)	*p*-Value	Holm-Adjusted *p*
A1 (Default)	SRT50	1.287	[1.270, 2.309]	*p* < 0.001	*p* < 0.001
A2 (DNN)	SRT50	0.799	[1.120, 3.085]	*p* < 0.001	*p* < 0.001
B1 (Default)	SRT50	1.353	[1.702, 2.999]	*p* < 0.001	*p* < 0.001
B2 (DNN)	SRT50	1.565	[4.601, 7.485]	*p* < 0.001	*p* < 0.001
C (Default)	SRT50	1.245	[4.464, 8.289]	*p* < 0.001	*p* < 0.001
D (Default)	SRT50	0.728	[1.404, 4.358]	*p* < 0.001	*p* < 0.001
E (Default)	SRT50	1.034	[2.162, 4.607]	*p* < 0.001	*p* < 0.001
A1 (Default)	SRT80	0.849	[0.804, 2.066]	*p* < 0.001	*p* < 0.001
A2 (DNN)	SRT80	1.672	[2.425, 3.820]	*p* < 0.001	*p* < 0.001
B1 (Default)	SRT80	0.745	[0.952, 2.865]	*p* < 0.001	*p* < 0.001
B2 (DNN)	SRT80	1.387	[4.601, 7.992]	*p* < 0.001	*p* < 0.001
C (Default)	SRT80	1.576	[4.995, 8.095]	*p* < 0.001	*p* < 0.001
D (Default)	SRT80	0.777	[1.304, 3.719]	*p* < 0.001	*p* < 0.001
E (Default)	SRT80	1.132	[2.108, 4.185]	*p* < 0.001	*p* < 0.001

## Data Availability

The data supporting the findings of this study are available from the corresponding author upon reasonable request, subject to applicable confidentiality and proprietary restrictions.

## Nomenclature

AEMT, American English Matrix Test; AI, artificial intelligence; ASR, automatic speech recognition; BSIM, binaural speech intelligibility model; BTE, behind-the-ear; ESTOI, extended short-time objective intelligibility; CI, confidence interval; DNN, deep neural network; HINT, Hearing in Noise Test; NLMS, normalized least-mean-squares; RIC, receiver-in-canal; SNR, signal-to-noise ratio; SPL, sound pressure level; SRT, speech reception threshold; SRT50, speech reception threshold corresponding to 50% correct word recognition; SRT80, speech reception threshold corresponding to 80% correct word recognition; STOI, short-time objective intelligibility; STT, speech-to-text.
